# Comparison of Radial Artery Patency Rates One Month Post-intervention: Distal vs. Proximal Radial Access for Coronary Interventions

**DOI:** 10.7759/cureus.78603

**Published:** 2025-02-06

**Authors:** Arcot Krishna Kishore, Varsha Rakshitha Prakash, Vadagenalli Sathyanarayana Rao Prakash, Neeraj Shivakumar

**Affiliations:** 1 Cardiology, Ramaiah Medical College and Hospital, Bengaluru, IND; 2 Radiology, Ramaiah Medical College and Hospital, Bengaluru, IND

**Keywords:** coronary artery disease, coronary intervention, distal radial artery access, proximal radial artery access, radial artery occlusion

## Abstract

Background

Traditionally, transfemoral and conventional trans-radial access sites have been used in coronary interventions. While the former is prone to complications like bleeding, hematoma, arteriovenous (AV) fistula, and increased morbidity and mortality, the latter is associated with instances of spasm and occlusion of the artery and compartment syndrome. Distal radial and ulnar access have recently been explored as alternative access sites. Therefore, this study was conducted to evaluate and compare its safety and efficacy with the conventional proximal radial access with a special focus on patency of the proximal radial artery at 30 days post-intervention and preserve proximal radial access for future coronary interventions.

Methodology

This prospective, randomized controlled study was conducted after obtaining approval from the Institutional Ethics Committee. A total of 150 patients suspected to have coronary artery disease and undergoing coronary interventions were included after obtaining voluntary, written, and informed consent, provided they met the inclusion and exclusion criteria. They were randomly divided into two groups: Group P (proximal radial access) and Group D (distal radial access [DRA]). Demographic details and relevant histories were noted. Under aseptic conditions, access was secured as per the assigned group. Intraoperative findings were noted. The patients were followed up on postoperative days 1, 7, and 30, and a Doppler study was done to assess for any occlusion at the proximal radial artery access site.

Results

In the present study, it was noted that both groups were similar in terms of demographic details and personal histories. The sheath insertion time (*P*-value = 0.039) and time to hemostasis (*P*-value < 0.001) were significantly less in Group D as compared to Group P. The number of attempts (*P*-value = 0.034) and the number of crossovers (*P*-value = 0.04) were higher in Group D as compared to Group P, with successful sheath insertion rates being higher in Group P (*P*-value = 0.04). Radial artery occlusion was significantly less in Group D as compared to Group P on days 7 and 30 (*P*-value = 0.004). However, the postoperative pain was significantly more in Group D (*P*-value < 0.001).

Conclusions

DRA is a safe and effective alternative to the conventional proximal radial access technique for coronary interventions. DRA is also useful as it preserves the patency of the proximal radial artery, which is useful for access during future interventions.

## Introduction

Cardiovascular diseases, which include coronary heart disease, peripheral vascular disease, heart failure, congenital heart disease, and cardiomyopathy, are the leading cause of disability and premature deaths globally [1], with coronary artery disease (CAD) causing 16.2% of all-cause deaths in 2019 [2]. Low- and middle-income countries account for 75% of these deaths [3]. Thus, with the rising incidence of CAD, the need for evaluation and coronary intervention is also on the rise. Traditionally, transfemoral access was the preferred modality.

Trans-radial access (TRA) for coronary angiography (CAG) was first performed in 1947 by Radner [4]. The first transluminal coronary angioplasty using TRA was done in 1992 by Kiemeneij and Laarman [5]. The trans-radial approach was associated with increased safety because of the reduction of major vascular complications and increased patient comfort, resulting from immediate post-procedural mobilization [5]. The 2018 European Society of Cardiology/European Association of Cardio-Thoracic Surgery recommended the radial artery as the preferred access site for any percutaneous coronary intervention (PCI) irrespective of clinical presentation unless there are overriding procedural considerations. It has hence been used widely and has become the gold standard approach for access in coronary interventions [6].

The RadIal Versus femorAL access for coronary intervention (RIVAL) trial was a major trial conducted on 7,021 patients with acute coronary syndrome (ACS). The trial results showed lesser major vascular complications that included retroperitoneal hematoma, pseudoaneurysm (requiring ultrasound compression or thrombin injection/surgical repair), large hematomas requiring prolonged hospitalization, arteriovenous (AV) fistulae, limb ischemia, damage to adjacent nerve and other surgical access site repair [7]. The trial conducted by Valgimigli et al. was another major trial that also showed a reduction in net adverse cardiovascular events when radial access was used for cardiac catheterization among patients with ACS as compared to the use of femoral access [8]. However, this approach is also prone to certain complications, the most common being occlusion of the artery, especially after repeated procedures, limiting its usability. Preserving radial artery patency is important as the presence of radial artery occlusion limits the use of the radial artery for potential future cardiac catheterizations or its use as a conduit in patients undergoing coronary artery bypass grafting (CABG) or for the creation of AV fistulae in patients with end-stage renal disease [9].

Therefore, there has been a search for safer alternatives. Over recent years, there has been growing interest in the distal radial artery access in the anatomical snuff box due to its comparatively superficial location and favorable branching pattern. Also, the use of distal radial artery access allows preservation of the proximal site for future interventions. As there are not many studies available on the safety and efficacy of this approach and the potential to preserve the proximal radial artery for future interventions, the present study was conducted.

## Materials and methods

**Figure 1:**
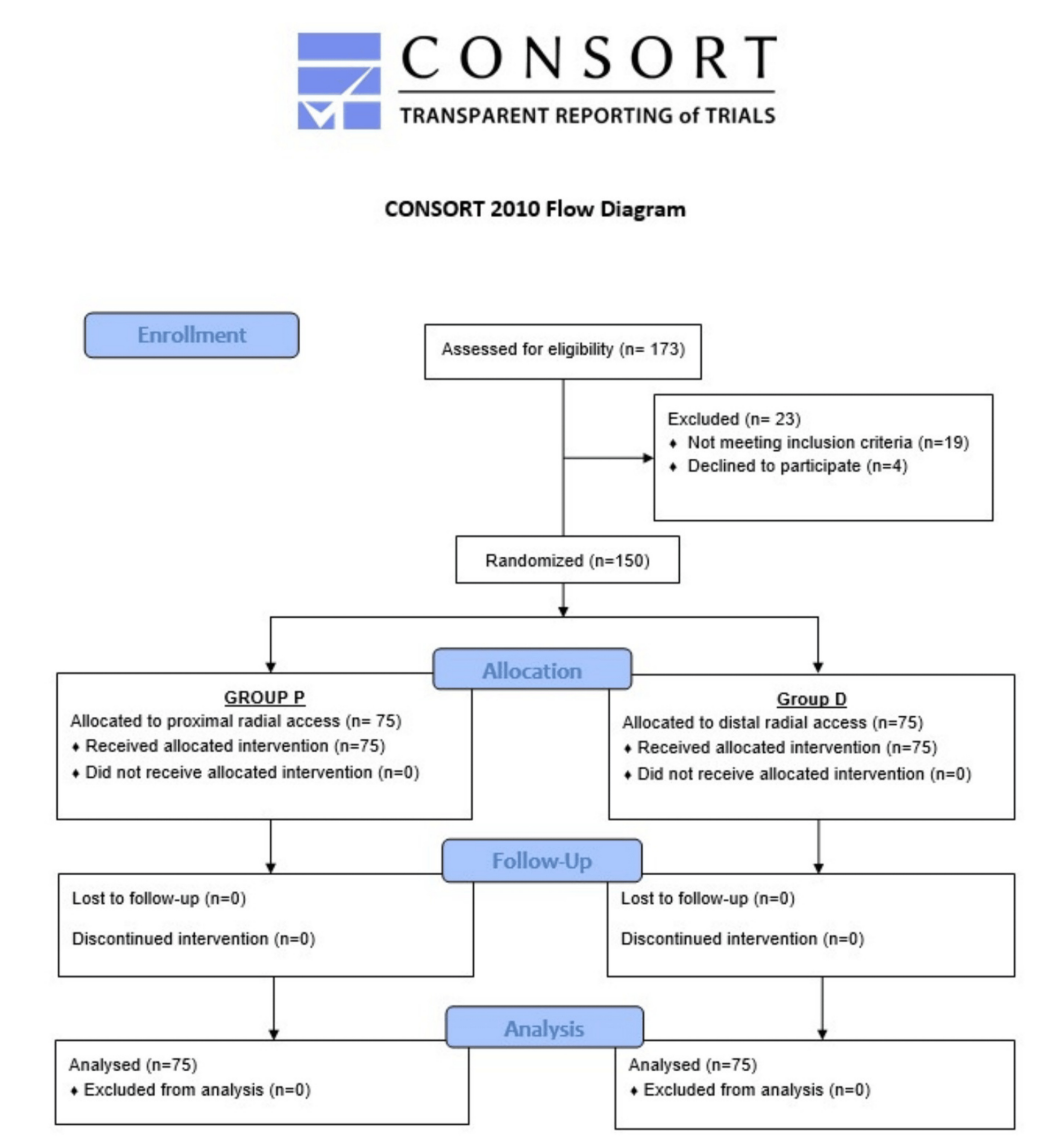
CONSORT 2010 flow diagram. CONSORT, Consolidated Standards of Reporting Trials

This prospective, randomized controlled study was conducted in the Department of Cardiology at Ramaiah Medical College, Bengaluru, after obtaining approval from the Institutional Ethics Committee. The study was conducted from February 20, 2024, to December 30, 2024. A voluntary, written informed consent was obtained from all the patients before their inclusion in the study. This study was conducted in accordance with the Consolidated Standards of Reporting Trials (CONSORT) 2010 guidelines (Figure 1) [10].

From the study by Das et al. to assess the usefulness of distal radial access (DRA) in the right anatomical snuff box for CAG and interventions, it was observed that 159 consecutive patients with a palpable right distal radial pulse prospectively at a single center were taken up and distal radial puncture was successful in 144 out of 159 (90.1%) patients [11]. In the present study, expecting similar results with 80% power and 95% confidence interval and considering the minimum detectable difference between the two methods, the study requires a minimum of 75 subjects in each group. A total of 150 patients aged 18 years and above and of either gender undergoing CAG or CAG with percutaneous transluminal coronary angioplasty (PTCA) were included in the study. Patients with fistula for hemodialysis, prior history of CABG, history of surgery at the access site or anatomical restrictions, patients with non-palpable radial artery, pregnant women, and those refusing to give consent to participate in the study were excluded.

Demographic details like age, gender, comorbidities, body mass index (BMI), indications for intervention, etc., were noted. Relevant past and personal histories were recorded. An electrocardiogram, echocardiography, cardiac enzymes, and other routine preoperative tests were done, and fitness was assessed. Patients were randomly divided into Group P and Group D. The patients in Group P underwent conventional proximal radial access, while those in Group D underwent DRA.

Procedure details

In the present study, right radial artery was used for arterial access as it is considered to be more convenient. As per European Society of Cardiology (ESC) and the Society for Cardiovascular Angiography and Interventions (SCAI) guidelines, the right side is ergonomically more suitable for majority of the operators [6,12].

Puncture site disinfection was performed, with shaving limited to the wrist quadrant.

The patient was positioned supine on the angiographic table with an arm board attached to the right-hand side of the table.

In Group P, the patient’s right arm was placed on the board and abducted at an angle of 30°, with the right wrist placed in a hyperextended position using a sandbag. Intravenous (IV) access and non-invasive blood pressure (NIBP) cuff were placed on the contralateral arm.

The access point was located on the radial artery 2 cm proximal to the styloid process. Lidocaine (1-2 mL) was administered as a local anesthetic. Using the through-and-through technique, arterial puncture was performed with a 20-gauge needle.

In Group D, the hand of the patient was positioned with the palm facing toward the midline of the patient with the anatomic snuffbox facing upward. Under aseptic precautions, the access point was located in the anatomical snuff box. After injecting the local anesthetic (1-2 mL Lidocaine), the arterial puncture was performed at a 30° to 45° angle in a lateral-to-medial direction.

Arterial access in both groups was obtained using the Seldinger technique.

As suggested by SCAI in their latest guidelines, hydrophilic sheaths were used due to their added benefits of less intimal trauma, increased patient comfort, and possibly higher long-term arterial patency [12]. After placement of the hydrophilic sheath - either the Prelude Ideal 6F hydrophilic sheath (Merit Medical, South Jordan, UT) or the 5F/6F Cordis sheath (Cordis, Miami Lakes, FL) - a *cocktail* was administered through it, consisting of 2,000 IU unfractionated heparin (UFH), 2.5 mg Verapamil, and 200 mg Nitroglycerin. In cases undergoing PTCA, an additional dose of UFH was administered to complete a total dose of 100 IU/kg.

Intraoperative characteristics of the time required for sheath insertion and time to hemostasis were noted. The number of attempts and crossovers required in each group were also noted. Pain was noted as per the visual analog scale (VAS) score [13].

In the postoperative period, patients were followed up on days 1, 7 and 30. At each follow-up visit, a Doppler study was performed to assess radial artery patency. Thrombus formation, if any, was noted.

Statistical analysis

Data were analyzed using SPSS software version 22.0 (IBM Corp., Armonk, NY). Categorical data were analyzed using the chi-square test (or Fisher’s exact test for cell values less than 5), while numerical data were analyzed using the Student’s t-test. A *P*-value of less than 0.05 was considered *statistically significant*.

## Results

The age distribution was similar in the two groups (*P*-value = 0.095). There was a male preponderance in both the groups, but the distribution was similar with *P*-value = 0.294. There was no significant difference in the distribution of BMI between the two groups (*P*-value = 0.294; Table 1).

**Table 1:** Distribution of demographic characteristics. BMI, body mass index

Parameter	Group P	Group D	*P*-value
Age	60.77 ± 10.84	63.57 ± 9.5	0.095
Gender (Male:Female)	48:27	54:21	0.294
BMI (kg/m^2^)	31.67 ± 4.09	30.99 ± 3.84	0.294

The two groups were similar in terms of the presence of comorbidities of hypertension and diabetes mellitus (*P*-value > 0.05). The two groups were also similar in terms of smoking and alcoholism (*P* > 0.05; Table 2).

**Table 2:** Distribution of comorbidities and personal history.

Parameter	Group P		Group D		Chi-square value	*P*-value
	n	%	n	%		
Hypertension	50	66.67%	53	70.67%	0.28	0.597
Diabetes mellitus	44	58.67%	46	61.33%	0.11	0.739
Smoking	46	61.33%	51	68.00%	0.73	0.393
Alcoholism	30	40%	34	45.33%	0.44	0.509

The majority of patients in both groups had single-vessel disease with similar distribution in the two groups (*P*-value = 0.236). When assessing procedure characteristics, the proportion of patients undergoing CAG with PTCA was significantly higher in Group P compared to Group D (*P*-value < 0.001). The number of attempts was more in Group D as compared to Group P (*P*-value = 0.034), and the number of crossovers was also higher in Group D (*P*-value = 0.04) (Table 3).

**Table 3:** Distribution of the disease and procedure characteristics. *Statistically significant *P*-value < 0.05. CAG, coronary angiography; PTCA, percutaneous transluminal coronary angiography

Parameter	Group P		Group D		Chi-square value	*P*-value
	n	%	n	%		
Multiple vessel disease	31	41.33%	24	32.00%	1.41	0.236
CAG with PTCA	47	62.67%	24	32%	14.15	<0.001*
More than one attempt	6	8%	15	20%	4.48	0.034*
Crossover required	5	6.66%	13	17.33%	4.04	0.04*
Successful sheath insertion	70	93.30%	62	82.66%	4.04	0.04*

Intraoperative procedure time, including sheath insertion time and time to hemostasis, was significantly shorter in Group D compared to Group P (*P*-value < 0.05). However, pain was higher in Group D (*P*-value < 0.001; Table 4).

**Table 4:** Distribution of procedure characteristics and pain scores. *Statistically significant *P*-value < 0.05. VAS, visual analog scale; SD, standard deviation

Parameter	Group P	Group D	*t*-value	*P*-value
	Mean ± SD	Mean ± SD		
Sheath insertion time (seconds)	142.40 ± 26.24	126.27 ± 61.40	2.09	0.039*
Time to hemostasis (minutes)	169.20 ± 14.50	129.60 ± 14.09	16.96	<0.001*
Pain by VAS score	2.21 ± 1.13	2.99 ± 1.25	-3.98	<0.001*

When assessed by the Doppler study during follow-up in the postoperative period, both groups were initially comparable. However, on Days 7 and 30, the proportion of cases with access site occlusion was significantly higher in Group P compared to Group D (*P*-value = 0.004; Table 5).

**Table 5:** Distribution of postoperative Doppler findings of the access site. Fisher's exact test was performed for parameters with cell values less than 5; therefore, the chi-square value is not available for these parameters. *Statistically significant *P*-value < 0.05. NA, not available

Parameter	Group P		Group D		Chi-square value	*P*-value
	n	%	n	%		
Access site occlusion on Day 1	16	21.33%	10	13.33%	1.67	0.196
Access site occlusion on Day 7	16	21.33%	4	5.33%	NA	0.004*
Access site occlusion on Day 30	16	21.33%	4	5.33%	NA	0.004*

## Discussion

In the present study conducted to evaluate the potential benefits of distal radial artery access for coronary interventions, sheath insertion time and time to hemostasis were found to be shorter with DRA compared to proximal radial access. Radial artery occlusion rates were significantly lower in the DRA group up to 30 days of follow-up after a trans-radial coronary procedure, as indicated by the Doppler study. However, the number of attempts was significantly higher in the DRA group, along with higher crossover rates. Pain was assessed using the VAS score and was observed to be higher with DRA.

In our study, the mean age of patients was 60.77 ± 9.50 years in the proximal radial group and 63.57 ± 9.50 years in the distal radial group, with a similar age distribution (*P*-value = 0.095). There was a male preponderance in both groups, with distribution being similar in the groups (*P*-value = 0.294). In the study by Vefalı and Sarıçam conducted on 205 patients in Ankara, they noted that the mean age of the patients in the traditional radial group was 59.84 ± 8.48 years and in the distal radial group was 60.89 ± 10.81 years [14]. They also noted a male preponderance in both groups. The age and gender distribution were similar between the two groups (*P*-value > 0.05).

In our study, the two groups were similar in terms of comorbidities such as hypertension and diabetes mellitus (*P*-value > 0.05). To eliminate confounding bias, we also compared smoking and alcoholism, with no significant difference between the groups (*P*-value > 0.05).

Similarly, in the study by Erdem et al., the groups were compared in terms of the presence of smoking, hypertension, diabetes mellitus, and hyperlipidemia, and there was no statistical significance [15].

In our study, intraoperative procedure time, including sheath insertion time and time to hemostasis, was compared between the groups. The distal radial group required significantly less time than the proximal radial group (*P*-value < 0.05). In the study by Vefalı and Sarıçam, the time to hemostasis was significantly shorter in the distal radial group (11.85 ± 1.91 minutes) compared to the traditional radial group (20.23 ± 4.19 minutes) (*P*-value < 0.0001) [14]. The findings were similar to the present study.

In the study by Erdem et al., the time to hemostasis was 33.3 ± 6.6 minutes in the distal TRA group and 43.9 ± 5.2 minutes in the conventional TRA group (*P*-value < 0.001) [15]. These findings were similar to the present study. However, they reported the sheath insertion time to be significantly more in the distal TRA group (3.2 ± 1.8 minutes) as compared to the conventional TRA group (1.5 ± 0.6 minutes) (*P*-value < 0.001) as opposed to the present study.

In our study, we observed that the pain compared between the two groups using the VAS score was significantly more in the distal radial group, with the *P*-value being <0.001.

In the study by Erdem et al. [15], they found the VAS pain score to be higher in the distal TRA group (3.48 ± 0.76) as compared to the conventional TRA group (2.98 ± 0.60) (*P*-value = 0.034), which was similar to the present study.

Studies have reported higher post-procedural pain after DRA as compared to proximal radial access. However, none of the studies have reported any disability or restriction of function following DRA.

In our study, crossover was required in five patients in the proximal radial group and 13 patients in the distal radial group, with a statistically significant difference (*P*-value = 0.04). In the famous distal vs. conventional radial access (DISCO Radial) Trial conducted by Aminian et al., the crossover rates were higher with DRA (3.5% vs. 7.4%, with *P*-value = 0.002) [16]. The findings were similar to the present study.

In our study, it was found that the radial artery occlusion upto day 30 was significantly less as found by radial artery Doppler in the distal radial group as compared to the proximal radial group.

The Distal Radial Artery Approach to Prevent Radial Artery Occlusion Randomized Controlled Trial (DAPRAO RCT) conducted by Eid-Lidt et al. demonstrated that radial artery occlusion rates were significantly lower in the DRA group at 24 hours (*P*-value = 0.015, risk reduction of 96%) and at 30-day follow-up (*P*-value = 0.047, risk reduction of 88%) [17]. Similar findings were observed in other studies and meta-analyses as well [18,19,20].

The crossover rates in the DAPRAO RCT were also similar to our study, with a higher crossover rate in the distal radial artery access group (13.3% vs. 0.7%; *P*-value = 0.0001) [17].

The lower radial artery occlusion rates in the DRA groups may be due to the anatomical location of the access, which is distal to the point of bifurcation of the artery in the deep palmar arch. Hence, the deep and superficial branches enable the preservation of the blood flow to the artery, thereby preventing spasms and occlusion [21,22]. The anastomoses in the wrist and hand have been shown to preserve flow to the forearm radial artery following simulated radial artery occlusion at the level of anatomical snuff box [23].

Access failure is hypothesized to result from the greater difficulty in securing radial artery access in the anatomical snuffbox (DRA) compared to the distal forearm (proximal access) [24]. However, in the present study, comparable success rates were found for both groups though the radial artery diameter was not evaluated.

All studies and meta-analyses have reported less time to hemostasis in the DRA as compared to the proximal radial access. There are no set guidelines for the procedure to be followed for hemostasis. Nevertheless, the superficial position of the artery at the distal access site as compared to the proximal access site along with its shorter diameter may be conducive to the application of pressure and proper transmission of direct pressure to the puncture site, aiding in shorter time to hemostasis. Early hemostasis also allows early mobilization of the patient, which, in turn, may aid in early hospital discharge. A shorter hemostasis time may also lower the incidence of radial artery occlusion in the postoperative period [21,22].

Limitations

The present study was a single-center study and was limited by outpatient department (OPD) attendance and patient admissions for CAD. Therefore, the results may not be generalized.

## Conclusions

The present study effectively concludes that distal radial access is a safe and effective alternative to traditional proximal radial access for patients undergoing CAG with or without PTCA, though higher postoperative pain may limit its overall efficiency. Nevertheless, there is the added advantage of preservation of the proximal site for future interventions. Therefore, further multicentric large-scale studies are recommended to substantiate the efficacy and provide recommendations for its use as an alternative to the conventional procedure on a routine basis.
